# Effects of stannous fluoride dentifrice on gingival health and oxidative stress markers: a prospective clinical trial

**DOI:** 10.1186/s12903-024-04785-7

**Published:** 2024-08-30

**Authors:** Niranjan Ramji, Sancai Xie, Ashley Bunger, Rachel Trenner, Hao Ye, Teresa Farmer, Tim Reichling, Julie Ashe, Kimberly Milleman, Jeffery Milleman, Malgorzata Klukowska

**Affiliations:** 1 The Procter & Gamble Company, Mason Business and Innovation Center, 8700 Mason-Montgomery Road, Mason, OH 45040 USA; 2 Salus Research Inc, 1220 Medical Park Drive, Building 4, Ft. Wayne, Fort Wayne, IN 46825 USA

**Keywords:** Stannous fluoride, Gingivitis, Oxidative stress, Clinical, Systemic health

## Abstract

**Background:**

Periodontal disease results in oral dysbiosis, increasing plaque virulence and oxidative stress. Stannous fluoride (SnF_2_) binds lipopolysaccharides to reduce plaque virulence. This study prospectively assessed SnF_2_ effects on oxidative stress in adults with gingivitis.

**Methods:**

This was a 2-month, single-center, single-treatment clinical trial. Twenty “disease” (> 20 bleeding sites with ≥ 3 pockets 3 mm-4 mm deep) and 20 “healthy” (≤ 3 bleeding sites with pockets ≤ 2 mm deep) adults were enrolled. All participants were instructed to use SnF_2_ dentifrice twice daily for 2 months. An oral examination, Modified Gingival Index (MGI) examination and Gingival Bleeding Index (GBI) examination were conducted at baseline, 1 month and 2 months. Gingival crevicular fluid (GCF), saliva, oral lavage and supragingival plaque were collected at each visit to evaluate: Endotoxins, Protein Carbonyls, L-lactate dehydrogenase (LDH), Ferric reducing antioxidant power (FRAP), Oxidized low density lipoproteins (oxi-LDL), IL-6 and C-reactive protein (CRP). A subset-analysis examined participants considered at higher risk of cardiovascular disease. Change-from-baseline analyses within each group were of primary interest.

**Results:**

The disease group showed statistically significant reductions in GBI at Month 1 (67%) and Month 2 (85%) and in MGI at Month 1 (36%) and Month 2 (51%) versus baseline (*p* < 0.001). At baseline, the disease group showed greater LDH in GCF and oxi-LDL levels in saliva versus the healthy group (*p* ≤ 0.01). Total antioxidant capacity (FRAP) in saliva increased versus baseline for the disease group at Months 1 and 2 (*p* < 0.05), and levels for the disease group were greater than the healthy group at both timepoints (*p* < 0.05). SnF_2_ treatment reduced endotoxins (lavage) for both disease and healthy groups at Month 2 (*p* ≤ 0.021) versus baseline. There was a reduction in oxidative stress markers, namely protein carbonyl in saliva, at Months 1 and 2 (*p* < 0.001) for both groups and a reduction in cytokine IL-6 (lavage) in the disease group at Month 2 (*p* = 0.005). A subset analysis of participants at higher coronary disease risk showed reductions in endotoxins in lavage, oxi-LDL, and CRP in saliva at Month 2 (*p* ≤ 0.04).

**Conclusion:**

SnF_2_ dentifrice use reversed gingival inflammation, suppressed endotoxins and reduced some harmful oxidant products in saliva and gingiva.

**Clinical trial registration:**

Clinicaltrials.gov NCT05326373, registered on 13/04/2022.

## Introduction

Gingivitis, inflammation of the gingiva, is caused by the buildup of bacterial dental plaque on the teeth and in the gingival sulcus. Left undisturbed, the bacterial populations in the plaque undergo maturation and become pathogenic, producing a variety of toxins that trigger an inflammatory response of the host [1]. The inflammatory responses of the gingiva are dependent upon detection of invading microorganisms and their metabolites. This takes place through the actions of several toll-like receptors (TLRs) [2]. TLRs are a family of transmembrane proteins widely expressed by eukaryotic cells that present the first line of immunological defense for the innate immune system [3]. TLRs can recognize bacterial virulence factors associated with gingivitis such as Lipopolysaccharides (LPS) and outer membrane vesicles (OMVs) produced by gram negative anaerobic bacteria and peptidoglycan (PGN), lipoteichoic acid (LTA), and teichoic acid (TA) from some gram-positive bacterial cell walls [3]. The best and most studied examples of TLRs include the selective expression of TLR2 and TLR4 by cells of mucosal epithelial sites such as the sulcus of the oral cavity [4]. Upon oral pathogen infection, TLR-dependent gingival inflammation causes an influx of neutrophils, monocytes, and lymphocytes to facilitate bacterial clearance [5]. TLRs and bacterial endotoxins then play a role in further activation of the neutrophils recruited to the site of the oral infection [6]. The neutrophils produce a number of substances designed to clear bacterial infections including the production of significant amounts of reactive oxygen species (ROS) [7, 8]. The imbalance between ROS and the antioxidant defense system is described as oxidative stress. Oxidative stress leads to damage to the periodontium as well as presenting a potentially important factor in numerous diseases [9–11].

Dentifrices containing high bioavailable levels of stannous fluoride (SnF_2_) have proven effective for the treatment and prevention of gingivitis [12, 13]. In addition to producing reductions in the amounts of dental plaque on teeth, previous laboratory research has shown that SnF_2_ reduces plaque microorganism pathogenicity by binding to LPS and LTA ligands blocking the reactivity of these to TLRs, including TLR2 and TLR4 [14]. These laboratory results were confirmed in a clinical study, where it was shown that SnF_2_ treatment resulted in reduction of endotoxin content and plaque virulence in subgingival plaque [15]. A significant increase in subgingival tin levels was detected 12 h post brushing with stannous fluoride dentifrice, suggesting the potential direct neutralization of these toxins from pathogenic plaque bacteria [16]. It was separately demonstrated that SnF_2_ can modify microbial metabolism, including the suppression of short chain fatty acids associated with tissue inflammation [17]. The combination of efficacy of SnF_2_ in reducing levels of clinical gingival inflammation and the unique antimicrobial mechanisms led us to theorize whether treatment with SnF_2_ could have measurable effects on oxidative stress to the gingiva. A reduction in oxidative stress could be important since the effects of ROS are proposed as important to the potential correlation of whole-body systemic effects induced by periodontal diseases [18].

The ROS that contribute to oxidative stress include singlet oxygen, hydroxyl radicals, superoxide and peroxides. While these species can be measured in live cells using fluorescent dye-based assays, their high reactivity and transient nature makes measurements in tissues and biofluids much more difficult. Oxidative stress is therefore often evaluated by measuring the oxidation *products* of various substrates including proteins, lipids, carbohydrates [19].

Given the evidence showing SnF_2_ binds lipopolysaccharides to reduce plaque virulence and improve gingival health, we hypothesized that SnF_2_ may also reduce oxidative stress. The aim of this 2-month clinical trial was to explore the effects of SnF_2_ dentifrice on oxidative stress markers in participants with and without gingivitis. The study also measured the effects of SnF_2_ antibacterial treatment on cytokines and levels of systemic markers of inflammation and included confirmatory assessments of SnF_2_ in reducing clinical gingivitis and suppressing endotoxin levels. Lastly, a subset-analysis was conducted among participants at higher risk of cardiovascular disease.

## Methods and materials

This was a single-center, single-treatment, clinical study of two months duration conducted at Salus Research Inc. in Ft. Wayne, Indiana. All participants signed informed consent and the protocol was reviewed and approved by the institutional review board (U.S.IRB2022SRI/03).

### Study participants

The target enrollment of 40 generally healthy adult participants, 18 years of age or older, with at least 16 natural teeth was to be split evenly into two groups distinguished by their number of bleeding sites and pocket depth tat screening and at baseline:

- “Healthy” group presenting with no more than 3 bleeding sites and with pockets less than or equal to 2 mm deep.
- “Disease” group presenting with more than 20 bleeding sites with at least 3 pockets greater than or equal to 3 mm but not deeper than 4 mm.

Other inclusion criteria were agreement to delay elective dentistry and to refrain from use of non-specified oral hygiene products during treatment periods. Exclusion criteria included self-reported pregnancy, inability to comply with study procedures, rampant or untreated caries, severe periodontal disease, fixed orthodontic appliances or attachments for aligner treatment, having a dental prophylaxis within 2 weeks of plaque sampling visits, taking antibiotics or using anti-bacterial oral care products within 2 weeks of plaque sampling visits, and/or any other disease or condition that may interfere with examination procedures or the participant’s safe completion of the study.

### Study design overview

Following their screening visit and agreement to participate, participants received detailed instructions for the remainder of study. For each clinic visit, site staff contacted participants, either by phone (primary) or by e-mail, and instructed them to refrain from brushing their teeth, eating, drinking, flossing, chewing gum, using breath mints, and using tobacco after their evening brushing and before the morning of their visit. Small sips of water were allowed after participants completed their at-home oral lavage collection and up to 45 min prior to the visit. The evening prior to sampling, participants were instructed to brush their teeth with their current oral hygiene products (baseline) or assigned product (months 1 and 2) and reminded of the standard drinking, eating, and tobacco restrictions prior to study visits. Participants collected lavage immediately upon waking in the morning using provided sampling kits. At the clinic, participants were first asked to provide an unstimulated saliva sample. Next, the Oral examination was performed followed by the Modified Gingival Index (MGI) exam [20]. A licensed dental professional then removed supragingival (gumline and interproximal) plaque using a sterile curette from the GCF identified teeth (preferably upper right and left premolars and molars-buccal side only). GCF samples were than taken at the tooth/gum interface (buccal surfaces only) using care to avoid contact with the oral soft tissues. Participants then received a Gingival Bleeding Index (GBI) exam [21]. At the baseline visit, treatment products were then distributed to participants. Participants were instructed on product use, and the first-time product use was done at the site. All general comments and Adverse Event (AEs), if any, were recorded. Clinic visits at Months 1 and 2 followed the same procedures as the baseline visit.

### Treatment

Following the baseline clinic visit, participants were provided a soft manual toothbrush (Oral-B® Indicator) and a bioavailable 0.454% stannous fluoride dentifrice (Crest® Pro-Health Sensitive and Enamel Shield). Toothpaste tubes were over-labeled so participants were blind to treatment. During the 2-month treatment period of the study, participants were instructed to brush thoroughly with the toothbrush and dentifrice for 1 min, twice daily (morning and evening), using enough of the provided toothbrush to cover the whole length of the toothbrush head.

### Sample collections and clinical evaluations

Participants received at-home oral lavage kits for each visit and were instructed to take a morning oral lavage immediately when waking by rinsing with 4 ml of water for 30 s and then expectorating into a tube. They were asked to store the sample in the freezer and return the sample in the provided cold pack/return kit at their scheduled visit. At the clinic visits, saliva samples were also collected. Participants filled a pre-labeled tube with approximately 3 ml of unstimulated saliva (no longer than 10-min collection time). The remainder of each sample was placed on wet or dry ice and stored long term at -70˚C.

### Clinical assessments

Safety was assessed by oral examination. The evaluation of oral soft and hard tissues was conducted via a visual examination of the oral cavity and perioral area utilizing a standard dental light, dental mirror, and gauze. The structures examined include the dentition and restorations, gingiva (free and attached), hard and soft palate, oropharynx/uvula, buccal mucosa, tongue, floor of the mouth, labial mucosa, mucobuccal/mucolabial folds, lips, and perioral area. Any abnormal findings were recorded.

The MGI measured the visual severity of gingivitis using a scoring system ranging from 0 (none) to 4 (severe) [20]. Following MGI assessment, GCF samples were collected from the right and left premolar and molars area (4 periopaper strips) and all pooled into one tube. Prior to sample collection, a licensed dental professional removed supragingival plaque with a curette/scaler. GCF samples were collected using a standard method using periopaper strips [22]. The orange plastic portion of the strip was cut and the absorbent portion was placed into a pre-labeled vial with 600 µl of buffer. Sites were only sampled once. The periopaper strip samples were immediately placed on dry ice until transferred into a -70 °C freezer and transported under dry ice to the P&G Mason Business and Innovation Center for analysis. A final examination was conducted to evaluate gingival bleeding after probing with mild pressure using the GBI [21]. Each of the 6 gingival areas (i.e., buccal, mesiobuccal, distobuccal, lingual, mesiolingual and distolingual) is scored approximately 30 s after probing as follows: 0 indicates absence of bleeding after 30 s, 1 indicates bleeding observed after 30 s, and 2 indicates immediate bleeding observed. Bleeding Scores were derived from the GBI scores at each site, with a value of 0 if the GBI score was 0, and a value of 1 if the GBI score was either a 1 or 2. A full mouth bleeding score was determined by summing the Bleeding Scores over all sites scored.

### Analytical procedures

Endotoxins: Limulus Amebocyte Lysate (LAL) measurements were done with the Endochrome-K™ traditional LAL kinetic chromogenic assay kit (Charles River; Lebanon, CT, USA). The assay detects LPS, which catalyzes the activation of a proenzyme in the modified LAL. The activated proenzyme then splits p-Nitroaniline (pNA) from the colorless substrate, Ac-Ile-Glu-Ala-Arg-pNA. The product pNA is photometrically measured at 405 nm. This assay was used to determine the activities of bacterial components to activate the proenzyme. Briefly, ninety-six-well microplates were first equilibrated in a heating block for 10 min at 37 °C. Salivary samples contain abundant materials that activate the proenzyme. To quantify LAL activities, we first ran a dose-curve of pooled salivary samples to identify the dose that places LAL activities in the middle of LPS standard curve. The pooled salivary samples were prepared by combining 2 μl samples from each sample in this study. Each dose was prepared by serial dilution. We found 0.042 μl of pooled salivary and 0.25 μl of pooled lavage generated LAL activity readings, which fitted within the middle of LPS standard curves in the LAL assay. Next, we diluted the samples serially and used 0.042 μl of salivary samples and 0.25 μl of lavage samples for the LAL assay. 50 µl each of standard or diluted salivary or diluted lavage sample was dispensed into the microplate wells. Then 100 µl LAL reagent was added to each well. Plates were shaken gently, and the absorbance was measured at 405 nm on Spectramax M3 plate reader (Molecular Device; Sunnyvale, CA, USA) for an hour at an interval of 1 min. The results were recorded as relative EU.

Total Antioxidant Capacity in Saliva – FRAP: Ferric Reducing Antioxidant power (FRAP) assay, a measure of antioxidant activity, was carried out with an Oxiselect Ferric Reducing Antioxidant Power assay kit (Cell Biolabs Inc; San Diego, CA, USA). The assay protocol was run per manufacturer’s instructions. Oral lavage was collected after participants swished with 4 ml of water. 75 μl of centrifuged oral lavage sample was used in the assay. The FRAP of each sample was expressed at mM of Fe^2+^/μl. The total FRAP in lavage was determined by multiplying FRAP value with the volume of expectorated lavage provided by each participant.

Protein Carbonyl: The protein carbonyl, which indicates oxidation of proteins, was determined using Protein Carbonyl Content Assay Kit (Sigma-Aldrich; St. Louis, MO, USA) protocol by using 100 μl of saliva sample supernatant after centrifugation at 4000 rpm for 20 min at 4°C. The concentration was expressed as nmoles/100 μl.

C-Reactive Protein (CRP), an indicator of systemic inflammation in the body that is associated with cardiovascular disease, was determined using the ELISA kit, Generation II (Salimetrics LLC; Carlsbad, CA, USA). The Assay was run per the protocol. Saliva samples were centrifuged at 1500 g for 15 min. Samples were diluted with 150 µl supernatant and 150 µl of CRP Generation II Sample Diluent. Concentration of unknown sample was determined from standard curve in pg/ml.

Oxidized Low density Lipoprotein (oxi-LDL), a risk factor for adverse cardiovascular events, was determined using the ELISA kit (Mercodia AB; Uppsala, Sweden)**.** Saliva samples were thawed in room temperature water for 10 -15 min. Samples were centrifuged at 2500 g, 4 °C for 10 min to remove cell debris and the clear supernatants were used in the assay. Neat undiluted saliva supernatants were used in the assay using 25 µl of sample. The concentration (in mM/L) of the unknown sample was determined from the standard plot.

Lactate dehydrogenase (LDH): LDH is a cytosolic enzyme present in gingival keratinocytes. It is released into saliva when the cell membrane is damaged. The CyQUANT LDH Cytotoxicity Fluorescence Assay (Thermo Fisher Scientific, Waltham, MA, USA) was used to determine levels of LDH in the GCF samples. Results were reported as mU/ml.

Cytokine measurement: The V-PLEX Proinflammatory Panel 1 Human Kit (Meso Scale Diagnostics; Rockville, MD, USA) was used to measure cytokines, a marker of inflammation. Fifty µl of oral lavage samples, calibrators or controls were first added to each well. The rest of the assay procedures were performed following manufacturer’s instruction. The concentrations reported were in pg/ml.

Researchers analyzing these objective markers were blind to participants’ healthy or disease status.

### Statistical analysis

The sample size was determined by logistical reasons. Number of bleeding sites was the primary endpoint and change from baseline analyses within each group were of principal interest. For clinical endpoints, including number of bleeding sites and MGI, a t-test was used to assess the difference between groups at baseline and mean comparisons to baseline for each group were performed using paired difference t-tests. For biomarker endpoints, data collected from saliva, GCF, and lavage samples were not normally distributed. Hence, a natural logarithm transformation was applied to all biomarker endpoints. For visit comparisons within each group, an Analysis of Covariance (ANCOVA) was done to analyze each endpoint/analyte using visit as a fixed effect with baseline adjustment. The geometric means based on back transformation and 95% confidence interval were reported. No adjustments were made for multiple comparisons. For FRAP, ANCOVA was done to analyze data by using group and baseline as fixed effects. Least Square Means were reported. Secondary analyses were conducted comparing groups using the ANCOVA method, with the efficacy end point as the response and baseline as the covariate. For subset analyses in participants at higher risk for cardiovascular disease, ANCOVA was done to analyze each analyte using visit as a fixed effect with baseline adjustment. The geometric means based on back transformation and 95% confidence interval were reported. No adjustment was made for multiple comparison.

All statistical tests were two-sided with a 5% level of significance.

## Results

Table 1 shows demographic characteristics of the study population. Forty-two participants were enrolled into the study and 39 were randomized to treatment. Two participants dropped due to enrollment quota being met, 1 had a screening failure, and 1 participant dropped after week 4 (lost to follow-up) resulting in 38 participants completing the study. Study participants’ age ranged between 25 – 73 years with a mean age of 52 years. There were 34 (87%) female and 5 (13%) male participants. Healthy and disease groups were balanced on age, ethnicity, race, sex, and smoking status (*p* ≥ 0.4658).

**Table 1:** Baseline demographic and clinical characteristics

Demographic/Statistic or Category	Healthy (*n* = 19)	Disease (*n* = 20)	Overall (*n* = 39)	*P*-value
**Age (Years)**				
Mean (SD)	51.00 (14.674)	52.95 (11.473)	52.00 (12.992)	0.6456^a^
Min.-Max	28—73	25—67	25—73	
**Ethnicity**				
Not Hispanic or Latino^b^	19 (100%)	20 (100%)	39 (100%)	
**Race**				
American Indian or Alaskan Native^b^	1 (5%)	1 (5%)	2 (5%)	0.4658^c^
Asian^b^	0 (0%)	1 (5%)	1 (3%)	
Black or African American^b^	2 (11%)	0 (0%)	2 (5%)	
Multiracial^b^	1 (5%)	0 (0%)	1 (3%)	
White/Caucasian^b^	15 (79%)	18 (90%)	33 (85%)	
**Sex**				
Female^b^	17 (89%)	17 (85%)	34 (87%)	1.0000^c^
Male^b^	2 (11%)	3 (15%)	5 (13%)	
**Smoking status**				
Yes^b^	1 (5%)	1 (5%)	2 (5%)	1.0000^c^
No^b^	18 (95%)	19 (95%)	37 (95%)	
**Clinical Characteristics**				
Bleeding sites, mean (SD)	2.00 (1.202)	33.10 (9.624)	17.95 (17.175)	< 0.001^d^
MGI score, mean (SD)	1.28 (0.320)	2.48 (0.359)	1.89 (0.692)	< 0.001^d^

^a^Two-sided ANOVA *p*-value for the group comparison

^b^The number (percent) of participants in each category

^c^Two-sided chi-square *p*-value for the group comparison

^d^Two-sided Fisher's exact test *p*-value for the group comparison

Clinical results for gingivitis are shown in Table 2, including change from baseline means for number of gingival bleeding sites and MGI scores for healthy and disease groups. The disease group showed significant reductions in the number of bleeding sites at 1 month (67%) and 2 months (85%) and in MGI scores at 1 month (36%) and 2 months (51%) compared to baseline (*p* < 0.001).

**Table 2:** Clinical results for number of bleeding sites and MGI score change from baseline

Group	Change from Baseline Mean^a^ (SD)	*P*-value^b^	% Change from Baseline^c^	Median	Min.-Max
**Number of Bleeding Sites**					
**Month 1**					
Healthy	0.42 (1.742)	0.306	27.78	0.00	-3.00 to 3.00
Unhealthy	22.50 (10.501)	< 0.001	67.62	21.50	0.00 to 40.00
**Month 2**					
Healthy	0.26 (1.790)	0.530	13.33	1.00	-3.00 to 3.00
Unhealthy	27.26 (8.157)	< 0.001	85.15	25.00	18.00 to 47.00
**MGI Score**					
**Month 1**					
Healthy	0.43 (0.275)	< 0.001	32.25	0.38	0.01 to 1.06
Unhealthy	0.89 (0.316)	< 0.001	35.98	0.84	0.47 to 1.58
**Month 2**					
Healthy	0.33 (0.367)	0.001	22.50	0.32	-0.23 to 1.11
Unhealthy	1.26 (0.329)	< 0.001	51.49	1.29	0.82 to 1.87

^a^Change from baseline = Baseline – Final

^b^Tests mean change from baseline versus 0 (2-sided paired-difference t-test)

^c^Percent Change from baseline = 100 x ((Baseline—Final)/Baseline)

A holistic measure of treatment effects on cumulative ROS included analysis of antioxidant power provided by the FRAP assay which was carried out in oral lavage for disease and healthy participants at baseline and following 1 and 2 months of SnF_2_ treatment. Inter-group comparisons are shown in Table 3. Mean FRAP significantly increased for the disease group following 1 and 2 months of SnF_2_ use (*p* ≤ 0.016) and was statistically significantly larger (*p* < 0.05) for the disease group compared to the healthy group at both timepoints.

**Table 3:** FRAP assessment of antioxidant capacity in oral lavage^a^

**Analyte/Matrix**	**Group**	**Baseline Adjusted Mean (SD)**	**Inter-Group Comparison at Baseline** ***p*** ** value** ^**b**^	**1 Month Adjusted Mean (SD)**	**Inter-Group Comparison at 1 Month** ***p*** ** value** ^**b**^	**Change from Baseline to 1 Month** ***p*** ** value** ^**c**^	**2 Month Adjusted Mean (SD)**	**Inter-Group Comparison at 2 Month** ***p*** ** value** ^**b**^	**Change from Baseline to 2 Month** ***p*** ** value** ^**c**^
FRAP	Healthy	13,121.26 (7534.48)	ns	12,707.82 (6977.51)	0.049	0.771	12,415.25 (6531.93)	0.047	0.697
	Disease	11,133.04 (7418.44)		15,649.96 (10,391.52)		0.016	15,618.04 (8926.18)		0.007

*ns* Not significant

*p* > 0.05

^a^ Model: score = baseline + group

^b^ 2-sided p value comparing group using analysis of covariance

^c^ Tests mean change from baseline versus 0 (2-sided paired-difference t-test)

A summary of key individual markers that were analyzed in saliva, lavage and GCF, including some individual ROS associated with inflammation, is shown in Table 4. A number of these markers showed statistically significant differences (*p* < 0.05) between healthy and disease groups as well as changes associated with SnF_2_ treatment at month 1 and 2.

**Table 4:** Analysis of various components associated with toxicity, inflammation and ROS activity in biofluids^a^

**Analyte/Matrix**	**Group**	**Baseline Geometric Mean (CI)** ^**b**^	**Group Comparison at Baseline P value** ^**c**^	**1 Month Geometric Mean (CI)** ^**b**^	**2 Month Geometric Mean (CI)** ^**b**^	***P*** **-value: Baseline vs. 1 Month** ^**d**^	***P*-value: Baseline vs 2 Month** ^**d**^
Endotoxin—Saliva	Healthy	25.55 (19.10, 34.17)	ns	16.52 (12.35, 22.10)	17.96 (13.43, 24.03)	0.038	0.092
	Disease	35.24 (26.41, 47.01)		24.17 (18.12, 32.25)	22.55 (16.77, 30.31)	0.069	0.035
Endotoxin—Lavage	Healthy	4.72 (3.16, 7.07)	ns	2.85 (1.90, 4.26)	2.26 (1.51, 3.38)	0.081	0.012
	Disease	5.15 (3.42, 7.75)		2.98 (1.98, 4.49)	2.56 (1.68, 3.90)	0.064	0.021
Protein carbonyls—Saliva	Healthy	1.02 (0.86, 1.20)	ns	0.44 (0.37, 0.52)	0.28 (0.23, 0.33)	< 0.001	< 0.001
	Disease	1.00 (0.84, 1.18)		0.60 (0.51, 0.72)	0.33 (0.27, 0.39)	< 0.001	< 0.001
IL-6—Lavage	Healthy	0.67 (0.41, 1.10)	ns	0.70 (0.43, 1.15)	0.56 (0.34, 0.92)	0.905	0.597
	Disease	1.68 (0.99, 2.86)		1.22 (0.72, 2.08)	0.55 (0.31, 0.96)	0.4	0.005
LDH—GCF	Healthy	8.65 (5.93, 12.61)	0.005	6.67 (4.57, 9.72)	6.22 (4.22, 9.18)	0.331	0.228
	Disease	25.84 (18.02, 37.06)		15.74(10.96, 22.53)	19.13 (13.22, 27.70)	0.055	0.249
CRP—Saliva	Healthy	688.02 (568.36, 832.88)	ns	504.87 (417.06, 611.16)	778.10 (642.77, 941.93)	0.026	0.365
	Disease	689.11 (575.85, 824.64)		598.67 (495.46, 723.39)	848.43 (702.16, 1025.17)	0.284	0.116
Oxidized LDL—Saliva	Healthy	1.93 (1.50, 2.47)	0.01	N A	2.03 (1.58, 2.60)	N A	0.777
	Disease	3.88 (3.03, 4.96)		N A	3.38 (2.62, 4.34)	N A	0.43

*ns* Not significant

*p* > 0.05

^a^ Model: score = baseline + visit

^b^ Least squares geometric mean and corresponding 95% confidence interval

^c^2-sided *p*-value comparing group at baseline

^d^2-sided *p*-value comparing visits within each group using analysis of covariance

Salivary endotoxin analysis (Table 4) revealed 38% numerically higher levels in the disease group versus the healthy group at baseline (35.24 versus 25.55; *p* > 0.05). SnF_2_ dentifrice treatment produced statistically significant reductions in saliva endotoxins versus baseline for the healthy group at month 1 (35%; *p* = 0.038) and the disease group at month 2 (36%; *p* = 0.035). Lavage analysis revealed ~ 9% elevated levels in the disease versus healthy group at baseline (5.15 versus 4.72; *p* > 0.05). Endotoxins in lavage were decreased for both disease and healthy groups reaching statistically significant reductions (*p* ≤ 0.021) of 52% for healthy and 50% for disease at month 2.

Cytokine expression was measured in lavage by multiplex array. While no significant changes (*p* > 0.05) in expression were observed for IL-10, INFg, IL-12p70, IL-13, IL-1β, IL-2, IL-4, Il-8 and TNF-α, interesting results were seen for IL-6. The disease group had 2.5 × the level of IL-6 at baseline versus the healthy group (1.68 versus 0.67; *p* > 0.05) and SnF_2_ treatment produced a statistically significant IL-6 reduction (*p* = 0.005) from baseline at month 2 for the disease group.

Oxidation products of proteins include protein carbonyls, which were sampled in saliva. Baseline levels of protein carbonyls did not differ numerically for disease versus healthy groups (*p* > 0.05). Nevertheless, SnF_2_ treatment produced highly statistically significant reductions (*p* < 0.001) in levels for both groups following 1 and 2 months of treatment. Numerical levels continued to decrease from month 1 to 2 for both disease and healthy groups.

LDH was analyzed in GCF. The disease group showed fourfold elevated levels compared to the healthy group (*p* = 0.005) at baseline. SnF_2_ dentifrice treatment did not affect LDH in the healthy group but in the disease group LDH was directionally reduced (*p* = 0.055) by 39% at month 1.

Measures of CRP and oxi-LDL were carried out in saliva samples. For CRP, disease and healthy groups showed numerically the same levels at baseline (*p* > 0.05). Levels of Oxi-LDL were two-fold higher for the disease than healthy cohort (*p* = 0.01) at baseline.

Review of the data suggested that a subset of participants presented with levels of CRP and oxi-LDL which would place them at higher risk for cardiovascular disease based on published literature values. An analysis was therefore carried out on these individuals for endotoxin, CRP and oxi-LDL. This subset analysis included 8 participants with CRP measuring over 2000 units from both the healthy and disease cohorts. A separate subset analysis was carried out on 8 individuals presenting with oxi-LDL > 6, likewise including selected healthy and disease participants. Both analyses are shown in Table 5. For participants with > 2000 units CRP at baseline, both endotoxin (44% at 1 month, 64% at 2 months) and CRP (43% at 1 month, 35% at 2 months) were substantially reduced following 1 and 2 months of SnF_2_ dentifrice use with 2 month results significant from baseline (*p* = 0.033 for endotoxin and *p* = 0.04 for CRP). Likewise for participants with Oxi-LDL levels > 6 at baseline, SnF_2_ use produced a reduction in salivary levels of 46% following 2 months (*p* = 0.009).

**Table 5:** Subset analyses in participants at risk for cardiovascular disease^a^

Participants with baseline CRP > 2000 (*N* = 8, 4 each from Healthy and Disease groups)					
				*p*-value baseline vs. 1 month^c^	*p*-value baseline vs. 2 month^c^
**Analyte**	**Baseline Geometric Mean (CI)** ^**b**^	**1 Month Geometric Mean (CI)** ^**b**^	**2 Month Geometric Mean (CI)** ^**b**^		
Endotoxin—Lavage	6.17 (3.19, 11.93)	3.41 (1.76, 6.59)	2.21 (1.14, 4.28)	0.2	0.033
CRP—Saliva	2934.78 (2222.28, 3875.72)	1658.30 (1232.85, 2230.56)	1910.24 (1420.16, 2569.46)	0.009	0.04
**Participants with baseline Oxi-LDL > 6 (***N*** = 8, 2 from Healthy and 6 from Disease group)**					
Oxi-LDL—Saliva	8.86 (6.52, 12.05)	NA	4.77 (3.51, 6.49)	NA	0.009

^a^ Model: score = baseline + visit

^b^ Least squares geometric mean and corresponding 95% confidence interval

^c^2-sided *p*-value comparing visits within each group using analysis of covariance

## Discussion

The pronounced reductions in gingivitis seen in this study were consistent with previous reports [12, 13] and suggest a potentially rich landscape for the evaluation of complementary effects in reducing molecular markers of inflammation and oxidative stress. To understand the mode of action, we were interested in confirming the effects of SnF_2_ treatment in blocking the pro-inflammatory effects of plaque bacteria through the analysis of endotoxins. Endotoxins were evaluated by the LAL assay and we observed a significant reduction in endotoxins in salivary lavage in both disease and healthy groups, and in saliva for the disease group, after 2 months. These results confirm previous research showing that Sn^2+^ shows strong binding with both LPS (endotoxin) and LTA [14]. Oral endotoxins are implicated not only in oral disease [23, 24] but play a role in systemic inflammation having been implicated in cardiovascular disease, Type 2 diabetes, and Alzheimer’s disease [24, 25].

Human saliva is rich in antioxidant compounds. The primary antioxidants include uric acid, albumin, ascorbic acid, glutathione, and antioxidant enzymes. Under oxidative stress, the antioxidants can be depleted. Antioxidant capacity can therefore be an indicator of oxidative stress [26]. The antioxidant *capacity* in biofluids is thus a holistic measure of oxidative stress in the tissues. Antioxidant capacity can be measured by FRAP test, which represents a holistic measure of antioxidant activity in biofluids. In this investigation, the method was used on samples collected from the oral lavage. In this assay, Ferric iron (Fe^3+^) is initially reduced by electron-donating antioxidants present within the sample to its ferrous form (Fe^2+^). The Fe^2+^ reacts with a colorimetric probe that was measured spectroscopically. The assay measures the ability of antioxidant to deactivate free radicals by single electron transfer mechanism. Samples are compared to the iron standard for determining antioxidant activity. Antioxidant activity in saliva of patients with periodontitis has been reported to be significantly lower than healthy participants [27]. In the present study, a significant increase in FRAP activity of oral lavage was observed in the disease group after 1 month and 2 months of SnF_2_ treatment over baseline. These results suggest that as treatments reduce oxidative stress and free radical activity in the mouth, the relative proportions of natural antioxidant present in saliva are restored with the reversal of gingivitis by SnF_2_.

The biology of ROS mediated protein damage is highly complex. The oxidized proteins are poorly handled by cells and can accumulate. The effect of such accumulation can lead to functional inactivation, which may be reversible or non-reversible, and an increased susceptibility of degradation by protease. One important oxidation marker that is well studied is protein carbonylation. Published data shows an increase in saliva protein carbonyls with age [28]. There is a correlation of saliva protein carbonyls to plasma protein carbonyls [29] and high levels of protein carbonyl groups have been observed in Alzheimer’s disease, rheumatoid arthritis, diabetes, sepsis, chronic renal failure, and respiratory distress syndrome [30, 31]. In this study, we examined protein carbonyls in saliva. The disease and healthy groups showed similar levels of protein carbonyls at baseline, however there was a highly significant reduction in protein carbonyls in both the disease and healthy groups at 1 month and 2 months compared to baseline with SnF_2_ treatment.

Plasma LDL have been implicated in cardiovascular disease. Elevated levels of plasma LDL deposit in arteries. Due to oxidative stress, they are oxidized to form oxi-LDL which initiates an inflammatory response triggering the formation of atherosclerotic plaque [32]. Published literature shows that plasma oxi-LDL is a strong predictor for acute coronary heart disease events [33]. Plasma oxi-LDL comes into saliva via GCF and a correlation has been reported between salivary and serum oxi-LDL levels [34, 35]. In this study we observed that the disease group had significantly elevated levels of oxi-LDL at baseline versus the healthy group. SnF_2_ treatment produced a small, non-significant reduction in oxi-LDL in the disease group. From literature reports, we were able to estimate the saliva oxi-LDL that puts participants at a higher risk of cardiovascular disease [34, 35]. Looking across all participants, we identified 8 participants who had high-risk levels (> 6 μm/l). We demonstrated that SnF_2_ dentifrice significantly reduced levels of oxi-LDL at 2 months compared to baseline in this subset of participants.

Measures of CRP were carried out in saliva samples. CRP is produced in the liver and plasma CRP levels rise when there is inflammation in the body. It has been shown that there is a longitudinal association of elevated CRP with systemic inflammation and cardiovascular disease risk [36–38]. CRP is excreted into the saliva via GCF and saliva levels have been suggested as surrogates for measures in plasma [39]. As with oxi-LDL, looking across all participants we identified 8 participants who had levels that puts them higher risk of CVD (> 2000 pg/ml, Range 2000–5400 pg/ml) [34, 40]. In this subgroup of participants, there was a significant reduction of CRP after 1 month and 2 months of SnF_2_ dentifrice use over baseline.

In addition to markers of oxidative stress, analysis was carried out on molecular signatures of inflammation including cytokines in lavage. Endotoxin activation of TLRs and the infection of the gingiva induces gingival fibroblasts to excrete a cascade of cytokines in infected tissues [41]. One of the most prominent is IL-6, which has been associated with periodontal disease progression in susceptible patients [41, 42]. Interesting findings were observed in this study for IL-6, which showed threefold concentrations in lavage of disease versus healthy groups at baseline (non-significant). The disease group showed decreases in IL-6 at 1 month and a 67% decrease at 2 months following SnF_2_ treatment (p = 0.005).

LDH is a ubiquitous enzyme that has the pivotal role in the clinical diagnosis of many disease processes [43]. LDH is present in the cytoplasm of the cells and is released into the extracellular environment upon lysis and cell death. LDH is known to catalyze the oxidative conversion of pyruvate to lactate and has been used as a marker of inflammation. Thus, LDH represents a marker of cell death and tissue breakdown and elevated levels often signify a disease process [44]. LDH has been found to correlate to bleeding on probing in gingivitis [45]. This study examined LDH in GCF. The significantly elevated levels of LDH in the disease group and the reduction of levels in this group following SnF_2_ dentifrice treatment complemented the reductions in clinical bleeding sites.

**Fig. 1:**
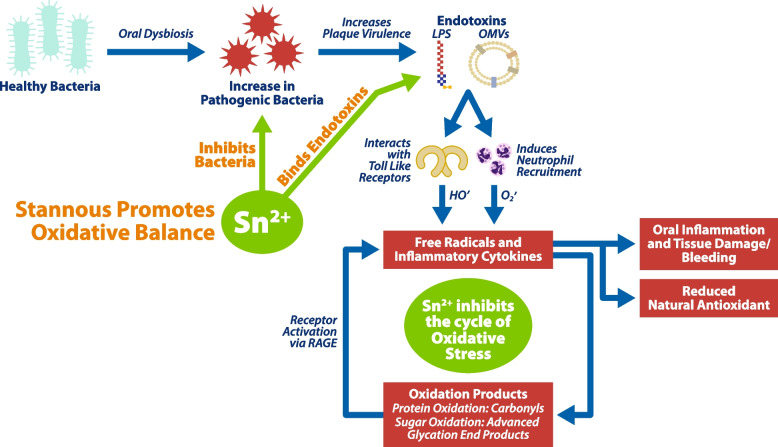
Proposed mode of action of SnF_2_ in reducing oral inflammation and reducing oxidative stress

This clinical trial investigated the potential effects of SnF_2_ antimicrobial dentifrice in reducing oxidative stress to the gingivae. To our knowledge, this is the first such evaluation reported in the literature. A reduction in oxidative stress could be important since the effects of ROS are proposed as important contributors to the potential systemic effects induced by periodontal disease [46]. We propose that SnF_2_ dentifrices have the potential to play a role in reducing systemic inflammatory burden in patients owing to downstream effects of their antibacterial activity in reducing the damage from ROS associated with oral inflammation. The proposed mechanism is diagrammatically shown in Fig. 1. Plaque bacteria undergo oral dysbiosis to mature into biofilms comprised of elevated populations of pathogens. The pathogens contain LPS (endotoxins) in their cell membranes and further secrete endotoxins as OMVs. These toxins interact with TLRs within the gingival sulcus (e.g., gingival fibroblasts) to both recruit neutrophils to the site of the infection and to directly promote ROS generation. Recruited neutrophils likewise interact with pathogen-generated LPS to further produce elevated levels of ROS [46]. All of this is part of the innate immune response effort to clear the bacterial infection [47]. The ROS help encourage tissue inflammation and the symptoms associated with gingivitis, including bleeding gums. The ROS generated in the inflammation produce a variety of oxidation products including protein carbonyls, oxidized lipids and oxidized sugars which further enhance inflammation. The inflammatory burden of the ROS are possibly an important contributor to the varied systemic conditions correlated with poor gingival health [11]. SnF_2_ can interrupt the cascade of effects of chronic inflammation by specific antibacterial effects in deactivation of endotoxin and modulation of bacterial metabolism.

The study results found here warrant further exploration of this mechanism. Longer duration studies may provide larger and more consistent changes in the measured markers of inflammation and ROS reactivity. Likewise, evaluations of treatment effects in populations with pronounced levels of periodontal disease would be useful. Lastly, it would also be worthwhile to compare various antimicrobial therapies for their effects on these parameters, given the apparent direct effects SnF_2_ would appear to have on the molecular basis for bacterial pathogenicity as well as modification of biofilm metabolic properties contributing to dysbiosis.

## Conclusion

The oral microbiome plays a critical role in oral health status. When gingivitis occurs, there is oral dysbiosis causing an inflammatory cascade that leads to oxidative stress, increasing the risk of more advanced periodontal disease with systemic implications. This 2-month clinical study demonstrated that the antibacterial properties of stannous fluoride dentifrice reduce gingivitis, endotoxins, and oxidative products*.*

## Data Availability

The datasets generated and analyzed during the current study are not publicly available as they are considered proprietary but may be available from the corresponding author on reasonable request.

## Abbreviations

AE: Adverse Event
ANCOVA: Analysis of Covariance
CRP: C-reactive protein
FRAP: Ferric reducing antioxidant power
GBI: Gingival bleeding index
GCF: Gingival crevicular fluid
LDH: L-lactate dehydrogenase
LAL: Limulus Amebocyte Lysate
LPS: Lipopolysaccharides
LTA: Lipoteichoic acid
LDL: Low density lipoproteins
MGI: Modified gingival index
OMVs: Outer membrane vesicles
oxi-LDL: Oxidized low density lipoproteins
pNA: P-Nitroaniline
PGN: Peptidoglycan
ROS: Reactive oxygen species
SnF_2_: Stannous fluoride
TA: Teichoic acid
TLRs: Toll-like receptors
